# A Case Report of Complex Clozapine Initiation Despite Contraindications

**DOI:** 10.1192/j.eurpsy.2023.2244

**Published:** 2023-07-19

**Authors:** G. Dumais-Lévesque, L. Béchard, E. Malenfant, M.-F. Demers, A.-P. Bouffard

**Affiliations:** 1 Psychiatry and Neurosciences, University Laval; 2 University Institute of Mental Health in Quebec; 3 Pharmacy Faculty, University Laval; 4 CERVO Research Center, Quebec, Canada

## Abstract

**Introduction:**

Clozapine is well known for its unique efficacy in treatment-resistant schizophrenia and to reduce violent behaviour. Unfortunately, life-threatening adverse reactions including ileus, myocarditis and agranulocytosis can hinder its use. In this context, some clinicians may be reluctant to initiate clozapine in patients who are prone to these adverse drug reactions.

**Objectives:**

To describe a complex clozapine initiation despite the presence of serious adverse effects and contraindications. The management of these adverse events, using effective multidisciplinary team leadership strategies, will also be described.

**Methods:**

A case report will be presented. The challenges faced while using clozapine and strategies implemented to pursue the use of this medication will be described.

**Results:**

A young black man with severe first episode psychosis was admitted to the early intervention outpatient clinic in Québec, Canada. Multiple aggression and critically disorganized behaviour prompted patient transfer to a specialized long-term care unit. Given the severity of the resistant disease and after a shared decision-making process with the family, clozapine was introduced despite ethnic neutropenia (down to 0,2 X 10^9^/L) and idiopathic cerebral lesions. Both gave rise to multiple concerns. A specific hematological surveillance protocol was designed. Facing multiple severe neutropenia episodes, the use of prophylactic granulocyte colony-stimulating factor (300 mcg SC weekly) was added after literature review and a favourable consult of both pharmacist and hematologist. Cardiac enzyme elevation also requested specialized investigation and follow-up. Specialized educators, social workers, and nursing all needed to be deeply involved in the treatment process and team coordination requested strong team building capacities. After 6 months, the patient is now taking clozapine 325 mg daily and his symptomatology has sufficiently reduced to allow hospital leave. The patient is now engaged in his recovery process.

**Conclusions:**

Using an evidence-based approach, promoting expertise from multiple healthcare professionals, and allowing a substantial amount of time to develop team cohesion were all crucial elements of this success story.

**Disclosure of Interest:**

None Declared

